# Data on biochemical indexes of HFD-fed mice treatment with metformin or resveratrol

**DOI:** 10.1016/j.dib.2016.07.049

**Published:** 2016-07-29

**Authors:** Wenjun Zhao, Aiyun Li, Xin Feng, Ting Hou, Kang Liu, Baolin Liu, Ning Zhang

**Affiliations:** a Experiment Center for Science and Technology, Shanghai University of Traditional Chinese Medicine, Shanghai 201203, China; b State Key Laboratory of Natural Medicines, Department of Pharmacology of Chinese Materia Medica, China Pharmaceutical University, Nanjing 211198, China

**Keywords:** Metformin, Resveratrol, High fat diet, Free fatty acids, Glycerol

## Abstract

To investigate the changes of physiological and biochemical indexes, male mice were fed a regular diet or short time high fat diet (HFD) for 10 days with oral administration of saline, metformin, resveratrol, or injected intraperitoneally (ip) with digoxin respectively every day. Food intake and body weight were recorded simultaneously. Blood was collected after mice were sacrificed and then tested with commercial kits. The data manifested that metformin and resveratrol only ameliorate free fatty acids and glycerol in HFD-fed mice. Data interpretation of this part can be found in the research article “Metformin and resveratrol ameliorate muscle insulin resistance through preventing lipolysis and inflammation in hypoxic adipose tissue” (Zhao et al.,) [1].

**Specifications Table**

**Table:** 

Subject area	*Biology*
More specific subject area	*High fat diet fed mice characterization*
Type of data	*Figure*
How data was acquired	*Food intake and body weight were recorded; biochemical indexes in serum were obtained with commercial kit and microplate reader*
Data format	*Analyzed*
Experimental factors	*Mice were fed a regular chow diet or HFD with oral administration of saline, metformin (200 mg/kg), resveratrol (50 mg/kg), or injected intraperitoneally (ip) with digoxin (1 mg/kg) respectively every day. Mice were fasted for 8 h before collecting blood from the orbital sinus.*
Experimental features	*Food intake and body weight were recorded. The measurement of the contents of free fatty acids (FFAs), glycerol, total cholesterol (TC) and triglyceride (TG) were performed using the commercial kits.*
Data source location	*Shanghai, China*
*Data accessibility*	*Data are presented in this article*

**Value of the data**

• The data of short-term high-fat fed mice shows some differences between the data of regular chow diet fed mice.
• The data demonstrates the different reason that caused tissue hypoxia [2].
• The data could give a basis for further experiments on revealing the underlying mechanisms of relative adipocyte hypoxia [3].

## 1 Data

**Fig. 1:**
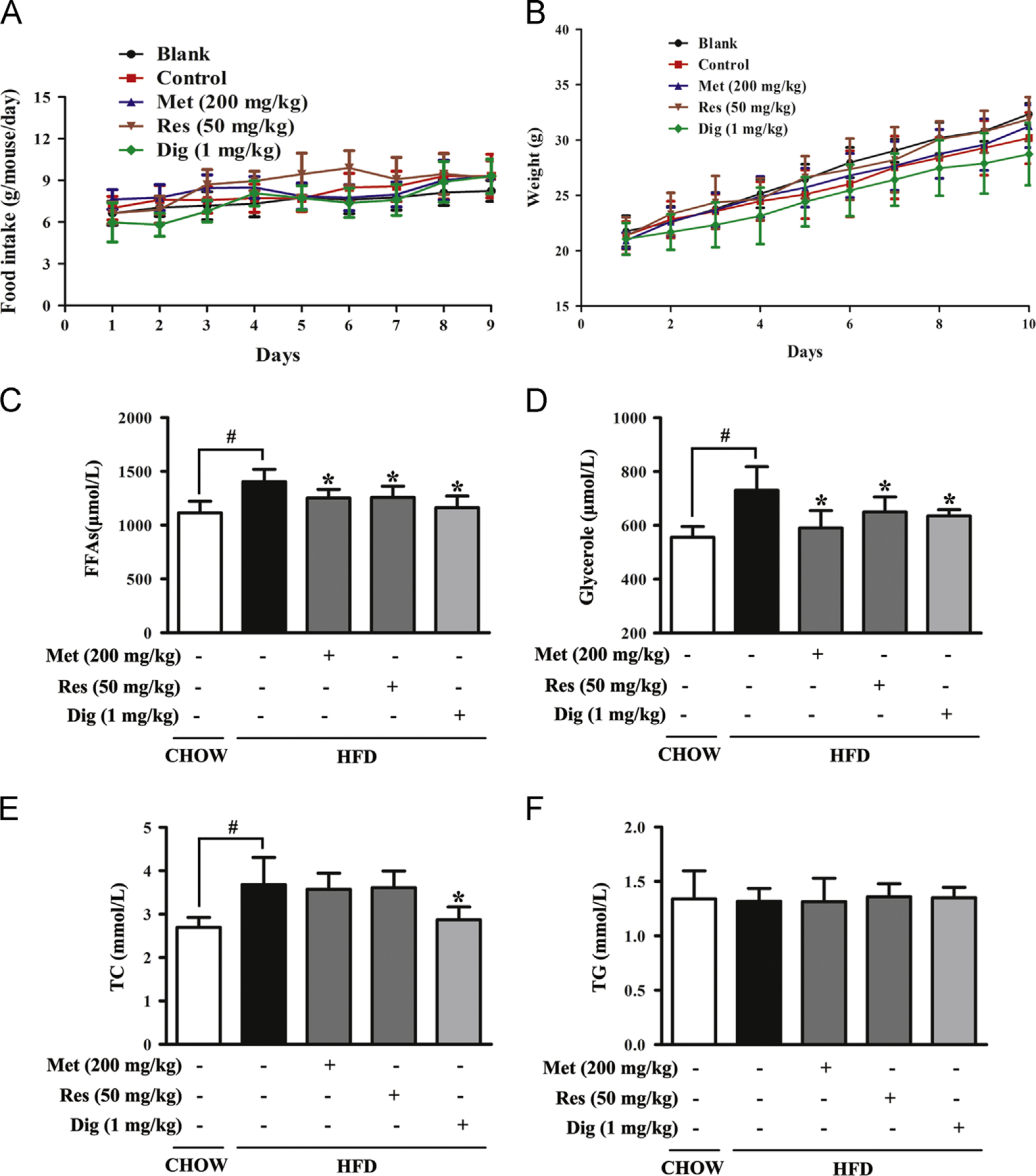
The effect of metformin and resveratrol treatment in HFD-fed mice.

This article provides the data changes in food intake, body weight and serum indexes of the HFD-fed mice (Fig. 1).

## 2 Experimental design, materials and methods

### 2.1 Experimental design and animals

The animals design was as the same as described in section of “animals” in [1]. Food intake and body weight were recorded.

### 2.2 Measurement of blood lipids

CHOW-fed and HFD-fed mice were fasted for 8 h before collecting blood from the orbital sinus. FFA (Jiancheng, Nanjing, China), Glycerol (Jiancheng, Nanjing, China), TG (BHKT, Beijing, China) and TC (BHKT, Beijing, China) levels were tested according to the manufacturer׳s instructions.

Mice were fed with HFD for 10 days with oral administration of metformin (Met, 200 mg/kg), resveratrol (Res, 50 mg/kg), or intraperitoneal injection of digoxin (Dig, 1 mg/kg, as the control reagent). (A) Food intake of per mouse every day, Data were expressed as the mean±SD (*n*=9). (B) Body weights of CHOW-fed and HFD-fed mice every day (mean±SD, *n*=10). (C, D, E, F) Serum were collected after mice were killed. Free fatty acid (FFA), glycerol, total cholesterol (TC) and triglyceride (TG) were measured by commercial assay kits (mean±SD, *n*=5–6). ^*^p<0.05 *vs* HFD feeding only treatment, ^#^*p*<0.05 *vs* the indicated treatment.
